# Splenic lymphangioma

**DOI:** 10.1016/j.ijscr.2019.07.078

**Published:** 2019-08-08

**Authors:** Thiam Ousmane, Faye Papa Mamadou, Sarr Ibrahima Sitor, Niasse Abdou, Dieng Madieng

**Affiliations:** a General Surgery Department at Dalal Jamm Hospital, Dakar, Senegal; b General Surgery Department at Aristide Le Dantec Hospital, Senegal

**Keywords:** Lymphangioma, Splenic, Rare

## Abstract

**Introduction:**

Abdominal localization of cyst lymphangioma is rare. The splenic involvement is exceptional.

**Case presentation:**

A 63-year-old woman, who was followed for martial anemia evolving associated with abdominal pain in the past 12 months. On clinical examination, she had pallor conjunctival mucosa, with a normal abdominal and lymph node examination. The abdominal ultrasound showed multiple splenic cysts. The abdominal CT scan showed a normal-sized spleen with multiples hypodense cystic lesions. At the laparotomy exploration a multinodular spleen was found which measured 18 cm*15 cm*6 cm. The histological exam results showed concluded to a splenic cavernous lymphangioma without malignity signs. The follow-up after 12 months was normal.

**Conclusion:**

splenic lymphangioma is rare and benign tumor. Total splenectomy under laparotomy or laparoscopy is the appropriate treatment.

## Highlights

• This is a case of abdominal cyst lymphangioma.
• In this pathology, the spllenic involvement is rare.
• The treatment is the splenectomy.
• The Histological exam confirms the diagnosis.

## 1 Introduction

Lymphangiomas are lymphatic vessels benign congenital malformations [1]. The head, neck and axillary regions are the common localizations. Intra-abdominal localization is rare and preferentially in the mesentery [2,3]. The rarity of lymphangiomas, the uncommon localization is a challenge for clinicians to make an accurate pre-operative diagnosis. Splenic lymphangiomas occur mainly in children and are exceptional in adult.

## 2 Case presentation

A 63-year-old woman, with hypertension for 12 years and under treatment (amlodipine and bisoprolol), who was followed for martial anemia evolving associated with abdominal pain, in the past 12 months, with oral iron treatment. Her pain was moderated and localized in the left upper quadrant without any radiation.

On clinical examination, she had normal vitals, pallor conjunctival mucosa, with a normal abdominal and lymph node examination. Her blood count revealed an anemia with 9,9 g/dl of hemoglobin.

The abdominal ultrasound showed multiple splenic cysts without ganglionic hypertrophy. The abdominal CT scan with intravenous contrast showed a normal-sized spleen with multiples hypodense cystic lesions without enhancement and no enlarged lymph nodes (Picture 1).

**Picture 1:**
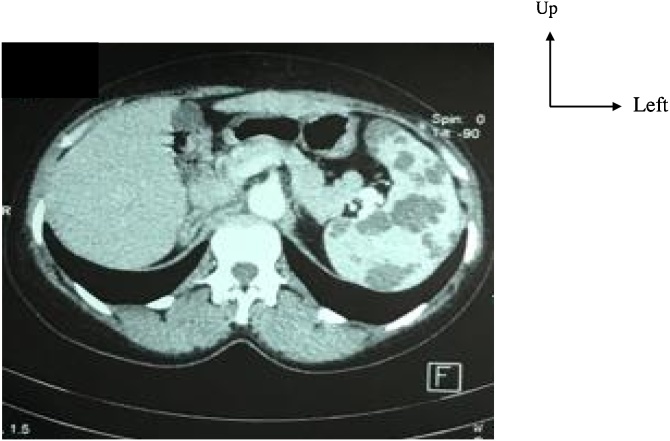
Abdominal CT scan showed multiples cystic lesions hypodense without enhancement. The spleen size is normal.

At the laparotomy exploration a multinodular spleen was found which measured 18 cm*15 cm*6 cm. The abdominal viscera were macroscopically healthy. A total splenectomy was done, with a simple post-operative period. She received pneumococcal, meningococcal and Hemophilus vaccination after the surgery.

The histological exam results showed a regular lymphoid splenic parenchyma with multiple cystic formations of variable diameter sometimes bordered by flattened endothelial cells. These cells are filled by serosities with lymphocytes and or red blood cells. There is fibrosis and calcification. It concluded to a splenic cavernous lymphangioma without malignity signs. The follow-up after 12 months was normal.

## 3 Discussion

Lymphangiomas are rare benign tumor of the lymphatic vessels, diagnosed the most in children and young adult [[4], [5], [6], [7]]. In 60% of cases, the diagnosis is made before 1 year old. The abdominal cystic lesions are preferably localized in the mesentery and the omentum. Splenic localization is exceptional [[8], [9], [10], [11]]. Most of the time asymptomatic, lymphangiomas can manifest itself by splenomegaly with left upper quadrant pain [12], or during complications like hemorrhage, coagulopathy, hypersplenism and poral hypertension [13,14].

The diagnosis is improved by medical imaging including ultrasound, CT scan and MRI [12]. Ultrasound is non-radiating, accessible and necessary in pre-natal diagnosis [15]. Ultrasound describes a hypoechoic splenic lesion with multiples septa and calcifications, not vascularized to Doppler [16,17]. The CT scan is radiating with more details on size of the spleen and the effects on the other organs [16]. The lesion at the CT scan is hypodense, homogeneous, with thin partitions and without intravenous contrast enhancement [17]. Partitions can be raised by the contrast if they are thick [18]. Magnetic resonance imaging has the same sensibility than the CT scan. Ultrasound and CT scan are the best imaging for the therapeutic strategy [19].

The differential diagnosis of splenic lymphangioma is broad including hemangioma, splenic infarction, septic embolism, lymphoma or metastasis [16]. There are three different types of splenic cystic lesions according to The Pearl-Nassar classification:

- Parasitic cyst with the dermoid, squamous or transitional epithelium,
- Cystic lymphangioma with a lymphatic endothelium
- Traumatic cyst without any coating [20].

In case of any doubt, a fine-needle aspiration cytology guided by ultrasound is performed. However, the incidence of false negatives varies between 0,06 % and 2% with a hemorrhagic risk [21,22].

The treatment of splenic lymphangioma depend of the lesion size and the presence of complications [16]. The treatment of choice is total splenectomy [23]. In per operative accessory spleens are removed. The laparoscopy has been proposed for the first time by Kwon in 2001 [24]. Many surgeons tried laparoscopy for the splenectomy [[25], [26], [27]]. However, splenectomy under laparoscopy has multiples contraindication including portal hypertension with high hemorrhagic risk and conversion, important splenomegaly (3,5 kg of weight, diameter superior to 20–25 cm) leaving insufficient space for pneumoperitoneum [[28], [29], [30]].

The surgery is performed without delays except in case of surinfection or other contraindications [16]. The rate of recidivism is low, even for the malignity risk. Few cases of malignant degeneration from lymphangioma to lymphangio-sarcoma was described [16]. The medical conservative treatment of splenic lymphangioma was described by Reinhardt and co. using the alpha interferon, in children with good tolerance [31]. However, the optimal dose and duration of treatment is not known, even if the treatment is curative [31].

The histological exam of the operative specimen allows to confirm the lymphangioma diagnosis. It shows cystic formation with septa constituted with a connective stroma with lymphoid tissue, striated muscle and lining with a lymphatic endothelium (positive factor D2-40) [15,[32], [33], [34]]. These results allow to eliminate parasitic cysts and to confirm the vascular origin of the tumor (Picture 2 ).

**Picture 2:**
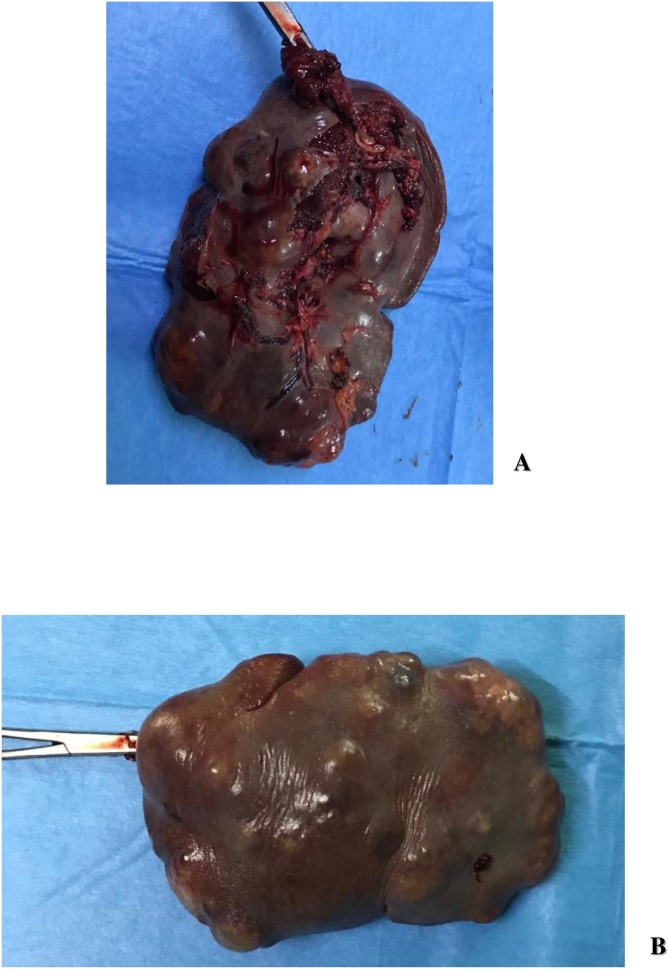
Operative piece A : posterior view B : anterior view.

## 4 Conclusion

Lymphangiomas are rare benign tumor of the spleen that are especially rare in adults. Splenic localization is incidental occurring in left upper quadrant pain exploration and rarely during a complication. The diagnosis is improved by the ultrasound and CT scan. But the histological exam allows to confirm the splenic lymphangioma diagnosis. The treatment is the total splenectomy under laparotomy or laparoscopy. The conservative medical treatment has not yet shown its effectiveness.

This manuscript was written according to the rules of the SCARE [35].

## Decleration of competing interest

The authors declare no conflict of interest.

## Funding

The authors declare they have received no funding for the preparation of this document

## Ethical approval

The ethical committee of the hospital gave the agreement to report this case.

## Consent

Written informed consent was obtained from the patient for publication of this case report and accompanying images.

## Author contribution

Thiam Ousmane, Faye Papa Mamadou these authors participated in the making and correction of this document. all authors agreed with the publication of the document.

## Registration of research studies

Researchregistr4759.

## Guarantor

Papa Mamadou FAYE.

## Provenance and peer review

Not commissioned, externally peer-reviewed.
